# Nerve Conduction Study in Healthy Elderly Subjects in Central India: A Cross-Sectional Study

**DOI:** 10.7759/cureus.28242

**Published:** 2022-08-21

**Authors:** Avinash B Taksande, Alka Rawekar, Sunil Kumar

**Affiliations:** 1 Physiology, Jawaharlal Nehru Medical College, Datta Meghe Institute of Medical Science, Wardha, IND; 2 Medicine, Jawaharlal Nehru Medical College, Datta Meghe Institute of Medical Science, Wardha, IND

**Keywords:** f-wave minimum latency, distal motor latency, effect of bmi on conduction velocity, conduction velocity, age, latency, central india, healthy elderly, nerve conduction

## Abstract

Background

There are many physiological changes that accompany aging. Slowing of muscle contraction, alteration of muscle metabolism and neuromuscular junction, and reduction of nerve conduction velocity (NCV) are among these physiological changes. The present study was conducted to elucidate the effect of physiological factors like gender, height, and Body Mass Index (BMI) on motor and sensory nerve conduction study (NCS) of the upper limb and find out the normal data for healthy elderly subjects in central India.

Methods

A cross-sectional study among 382 healthy adult participants of central India aged 60 years and above. The study was carried out in the department of Physiology, Acharya Vinoba Bhave Rural Hospital, Wardha, India, from July 2017 to June 2022. An NCS was performed using the Neuron Spectrum 5 machine (Neurosoft, Ivanovo, Russia). A Microsoft Excel spreadsheet (Microsoft Corporation, Redmond, Washington, United States) was used to tabulate the information gathered. For statistical analysis, IBM SPSS Statistics for Windows, Version 22.0 (Released 2013; IBM Corp., Armonk, New York, United States) was used.

Results

All NCS parameters were greater in males as compared to females. As age advances, longer distal motor latency (DML) and F-wave minimum latency, decreasing amplitude, and slowing of conduction velocity (CV) were observed. As height increases, increasing DML and F-wave minimum latency, decreasing amplitude, and slowing of CV were observed. Higher BMI was found to be associated with lower amplitudes and slowing of CVs.

Conclusion

Age and height have a negative influence on amplitude and CV is a positive influence on DML and F-min latency. Higher BMI has a negative influence on amplitude and CV.

## Introduction

Electrical pulses applied to the skin overlying peripheral nerves are usually sufficient to bring them to an action potential [1]. An NCS and needle electromyography (EMG) can be used to supplement the clinical examination of the peripheral nervous system [2]. It can help distinguish between neuropraxia and axonal transection in patients with extremity trauma and track their clinical progress [3]. There are many physiological changes that accompany aging. The slowing of muscle contraction, alteration of muscle metabolism and neuromuscular junction, and reduction of NCV are some of these physiological changes [4-6]. Neurological illnesses in older adults are a major cause of disability and dependence and account for a disproportionate number of patients treated in neurological outpatient and hospital settings [7]. Therefore, this study aims to elucidate the effect of these physiological factors (gender, height, and BMI) on motor and sensory nerve conduction study of the upper limb and find out the normal data for healthy elderly subjects in central India.

Objectives

To establish NCV values of the commonly tested upper limb nerves and to study the effects of gender, height, and BMI on NCV in healthy elderly subjects.

## Materials and methods

This is a cross-sectional study conducted at the Department of Physiology, Acharya Vinoba Bhave Rural Hospital, Wardha, India, between July 2017 and June 2022. Adults over the age of 60 who were otherwise healthy were recruited for the study.

Individuals over the age of 60 years make up 7.7% of India's population [8], and a margin of error of 5% was used to arrive at the sample size we used in the study. No previous history of trauma, neurological deficiency, or systemic illness that could cause neuropathy was found in the study's participants. Medical history was taken from each participant. Cases that met our inclusion criteria were included in the study after signing a written informed consent form. Parameters such as distal motor latency (DML), compound muscle action potential (CMAP) amplitude, and CV of the motor nerve and sensory nerve action. potential (SNAP) amplitude and CV for the sensory nerves as well as the motor nerve's shortest possible F-wave have been used to establish norms. An investigation into the transmission of nerve impulses was carried out with the Neuron Spectrum 5 apparatus ( Neurosoft, Ivanovo, Russia). A Microsoft Excel spreadsheet (Microsoft Corporation, Redmond, Washington, United States) was used to tabulate the information gathered. For statistical analysis, IBM SPSS Statistics for Windows, Version 22.0 (Released 2013; IBM Corp., Armonk, New York, United States) was used. For comparison, Chi-squared and Fisher's exact tests were used.

## Results

Demographic characteristics of the study population

Table 1 shows that among participants, the majority were in the age group of 60-65 years (35.34%) with a mean age of 68.43 ±5.67 years. The majority of the participants were male (82.46%) as compared to females (17.54%). 

**Table 1:** Demographic variables among participants

Variables		No. of participants (n=382)	Percentage
Age group (years)	60-65	135	35.34
	66-70	108	28.27
	71-75	88	23.04
	76-80	48	12.56
	>80	03	0.78
Gender	Male	315	82.46
	Female	67	17.54

Electrophysiological distribution

Table 2 shows normative data of NCS for median and ulnar nerves (motor and sensory) in the form of distal motor latency (DML), amplitude, CV, and F minimum latency.

**Table 2:** Electrophysiological distribution among the participants

Nerves	Parameters	Mean ±SD
Median nerve	Distal latency (msec)	3.91 ±0.76
	Amplitude (mV)	12.68 ±3.81
	Nerve conduction velocity (m/s)	54.66 ±5.87
	Sensory amplitude (µ)	32.17 ±14.43
	Sensory nerve conduction velocity (m/s)	57.88 ±6.83
	F minimum latency (msec)	27.61 ±3.73
Ulnar nerve	Distal latency (msec)	2.75 ±0.67
	Amplitude (mV)	12.88 ±2.86
	Nerve conduction velocity (m/s)	58.16 ±4.91
	Sensory amplitude (µ)	25.88 ±13.71
	Sensory nerve conduction velocity (m/s)	57.72 ±7.76
	F minimum latency (msec)	27.70 ±3.16

Electrophysiological distribution according to gender

Table 3 shows the electrophysiological distribution according to gender for the median and ulnar nerve.

**Table 3:** Electrophysiological distribution according to gender DML: distal motor latency; AMP: amplitude; CV: conduction velocity; S-AMP: sensory amplitude; S-CV: sensory conduction velocity; F min: F minimum latency

Nerves	Parameters	Male	Female	P-value
Median nerve	DML (msec)	3.91 ±0.74	3.90 ±0.84	0.92
	AMP (mV)	12.74 ±3.84	12.41 ±3.70	0.52
	CV (m/s)	54.80 ±5.90	53.99 ±5.80	0.31
	S-AMP (µ)	30.48 ±12.47	40.63 ±19.50	<0.0001*
	S-CV (m/s)	58.20 ±6.84	56.59 ±6.39	0.08
	F min (msec)	27.76 ±3.33	26.74 ±5.11	0.04*
Ulnar nerve	DML (msec)	2.69 ±0.63	3.02 ±0.82	<0.001*
	AMP (mV)	12.91 ±2.98	12.87 ±2.09	0.92
	CV (m/s)	58.01 ±4.81	58.96 ±5.31	0.15
	S-AMP (µ)	24.35 ±11.83	33.19 ±18.91	<0.0001*
	S-CV (m/s)	57.36 ±7.67	59.69 ±7.72	0.02*
	F min (msec)	27.97 ±3.02	26.38 ±3.45	<0.001*

Electrophysiological distribution according to age

Table 4 shows the electrophysiological distribution according to age for the median and ulnar nerve.

**Table 4:** Electrophysiological distribution according to age DML: distal motor latency; AMP: amplitude; CV: conduction velocity; S-AMP: sensory amplitude; S-CV: sensory conduction velocity; F min: F minimum latency

Nerves	Parameters	60-65	66-70	71-75	76-80	>80	P-value
Median nerve	DML (msec)	3.79 ±0.71	3.85 ±0.71	3.90 ±0.75	4.24 ±0.83	5.51 ±0.86	<0.001
	AMP (mV)	14.05 ±3.96	13.09 ±3.37	10.73 ±3.11	11.34 ±3.56	12.73 ±8.33	<0.001
	CV (m/s)	55.31 ±5.78	54.39 ±6.21	53.56 ±5.79	54.88 ±5.12	56.18 ±3.63	0.26
	S-AMP (µ)	37.43 ±14.70	32.39 ±11.86	27.46 ±14.64	26.90 ±13.57	9.99 ±3.60	<0.001
	S-CV (m/s)	59.51 ±7.44	57.33 ±6.12	57.19 ±6.31	55.98 ±6.79	54.88 ±7.33	0.008
	F min (msec)	26.75 ±2.90	27.05 ±2.95	27.05 ±2.95	29.19 ±5.25	25.73 ±1.15	<0.001
Ulnar nerve	DML (msec)	2.86 ±0.74	2.68 ±0.66	2.65 ±0.54	2.73 ±0.70	2.98 ±0.96	0.13
	AMP (mV)	12.88 ±3.01	12.98 ±3.10	12.83 ±2.70	12.74 ±2.25	13.87 ±1.32	0.96
	CV (m/s)	58.48 ±5.12	58.34 ±4.44	57.81 ±4.96	57.58 ±5.29	56.40 ±5.99	0.69
	S-AMP (µ)	29.04 ±15.29	26.03 ±12.31	22.45 ±12.89	23.88 ±11.44	10.77 ±4.76	0.001
	S-CV (m/s)	59.17 ±6.50	56.67 ±6.70	56.69 ±7.84	57.89 ±11.85	57.97 ±3.50	0.08
	F min (msec)	29.97 ±2.63	27.27 ±2.77	28.52 ±3.35	29.33 ±4.06	25.83 ±1.17	<0.001

Electrophysiological distribution according to height

Table 5 shows the electrophysiological distribution according to height for the median and ulnar nerve.

**Table 5:** Electrophysiological distribution according to height DML: distal motor latency; AMP: amplitude; CV: conduction velocity; S-AMP: sensory amplitude; S-CV: sensory conduction velocity; F min: F minimum latency

Nerves	Parameters	≤150	151-160	161-170	171-180	>180	P-value
Median nerve	DML (msec)	4.10 ±0.94	3.83 ±0.80	3.88 ±0.71	4.01 ±0.77	3.74 ±0.60	0.36
	AMP (mV)	12.58 ±4.67	12.35 ±3.67	12.61 ±4.01	12.99 ±3.43	13.74 ±4.23	0.72
	CV (m/s)	54.39 ±5.91	54.02 ±5.82	54.77 ±5.74	55.43 ±6.30	52.05 ±4.12	0.32
	S-AMP (µ)	39.23 ±24.18	37.39 ±17.59	31.27 ±11.86	27.77 ±11.92	27.97 ±11.92	<0.001*
	S-CV (m/s)	58.03 ±6.22	57.24 ±6.23	58.33 ±6.49	57.45 ±8.37	59.21 ±3.30	0.68
	F min (msec)	27.38 ±3.47	27.06 ±4.99	27.42 ±3.27	28.45 ±2.91	28.74 ±4.01	0.09
Ulnar nerve	DML (msec)	3.07 ±0.54	2.88 ±0.85	2.70 ±0.65	2.64 ±0.49	2.88 ±0.65	0.03*
	AMP (mV)	12.85 ±2.74	13.23 ±2.29	12.88 ±2.98	12.57 ±3.22	12.48 ±2.19	0.63
	CV (m/s)	55.10 ±3.35	58.63 ±5.28	58.57 ±4.65	57.96 ±5.10	53.15 ±0.68	0.001*
	S-AMP (µ)	30.78 ±17.66	31.07 ±18.54	25.16 ±10.95	21.45 ±10.37	22.48 ±8.49	<0.001*
	S-CV (m/s)	59.28 ±5.54	58.26 ±7.35	57.11 ±7.22	58.20 ±9.71	56.81 ±3.10	0.62
	F min (msec)	27.05 ±3.07	26.96 ±3.88	27.66 ±3.15	28.45 ±2.13	29.60 ±1.23	0.006*

Electrophysiological distribution according to BMI

Table 6 shows the electrophysiological distribution according to BMI for the median and ulnar nerve.

**Table 6:** Electrophysiological distribution according to BMI DML: distal motor latency; AMP: amplitude; CV: conduction velocity; S-AMP: sensory amplitude; S-CV: sensory conduction velocity; F min: F minimum latency

Nerves	Parameters	<18.5	18.5-24.9	≥25	P-value
Median nerve	DML (msec)	3.75 ±0.75	3.95 ±0.76	3.87 ±0.70	0.10
	AMP (mV)	13.35 ±4.12	12.62 ±3.64	11.09 ±4.15	0.02*
	CV (m/s)	54.44 ±5.30	54.74 ±6.07	54.55 ±5.84	0.83
	S-AMP (µ)	35.72 ±14.48	30.74 ±13.88	34.35 ±17.71	0.01*
	S-CV (m/s)	57.49 ±6.35	58.08 ±7.03	57.28 ±6.55	0.69
	F min (msec)	27.82 ±3.66	27.40 ±3.69	28.76 ±4.09	0.58
Ulnar nerve	DML (msec)	2.81 ±0.62	2.72 ±0.69	2.81 ±0.69	0.73
	AMP (mV)	13.34 ±3.03	12.85 ±2.87	11.94 ±1.98	0.06
	CV (m/s)	57.63 ±5.11	58.43 ±4.94	57.33 ±4.01	0.58
	S-AMP (µ)	28.71 ±16.14	24.87 ±12.69	26.55 ±13.80	0.07
	S-CV (m/s)	56.46 ±7.13	58.09 ±7.95	58.09 ±7.72	0.51
	F min (msec)	27.87 ±3.25	27.60 ±3.10	28.08 ±3.75	0.64

## Discussion

Clinical evaluations of individual patients and epidemiological studies rely on normal values. The present cross-sectional study was carried out to generate comparative normative data on NCS of healthy elderly subjects in central India in the tertiary care center. The findings of the NCV in the upper limb of the present study was shown in Table 7.

**Table 7:** Values of NCS in upper limb DML: distal motor latency; AMP: amplitude; CV: conduction velocity; S-AMP: sensory amplitude; S-CV: sensory conduction velocity; F min: F minimum latency; NCS: nerve conduction study Shehab [9]; Thakur et al. [10]; Palve and Palve [11]

Nerves	Parameters	Shehab et al.	Thakur et al.	Palve et al.	Present study
Median nerve	DML (msec)	3.1+0.3	2.68±0.29	3.95 ±0.6	3.91 ±0.76
	AMP (mV)	11.1+2.8	9.68±3.11	-	12.68 ±3.81
	CV (m/s)	56.5+3.5	-	56.7 ±1.1	54.66 ±5.87
	S-AMP (µ)	-	-	-	32.17 ±14.43
	S-CV (m/s)	-	-	54.5 ±7.5	57.88 ±6.83
	F min (msec)	-	23.82±1.48	25.3 ±1.6	27.61 ±3.73
Ulnar nerve	DML (msec)	2.4+0.3	2.25±0.54	-	2.75 ±0.67
	AMP (mV)	9.2+2.2	9.09±2.65	-	12.88 ±2.86
	CV (m/s)	60.4+5.2	-	-	58.16 ±4.91
	S-AMP (µ)	-	-	-	25.88 ±13.71
	S-CV (m/s)	-	-	-	57.72 ±7.76
	F min (msec)	-	24.88±1.79	-	27.70 ±3.16

The mean age among male and female subjects was 68.33 ±5.69 and 68.85 ±5.08 years, respectively. The findings showed that significantly longer DML was observed as age advances except for ulnar DML. There was a clear decline in CMAP amplitude as well as SNAP amplitude as a person gets older. This decline is significant in the motor median nerve. Slowing of motor and sensory CV was observed in the older age group. The slowing is significant in sensory ulnar and sensory median nerves. F-wave demonstrated a significant (p<0.05) rise in F-wave minimum latency as age progressed. Research has shown that CV begins to decline around the age of 30-40 years, but by the time people reach their 60s or even their 80s, the values dropped by about 10 m/s. The loss of myelinated and unmyelinated nerve fibers in peripheral nerves with aging may be responsible for the decline in nerve conduction and the increase in sensory latency that occurs with age [9-11]. The effect of sex on NCS can be explained on the basis of gender-wise differences in anatomical and physiological factors [12]. We observed greater values of SNAP amplitude and sensory CV in females as compared to males, especially in ulnar nerves. This might be because of sampling error (less number of females). Robinson et al. [13] explained that gender differences in nerve conduction parameters could be due to differences in height.

In the present study, the majority of subjects were in the height group 161-170 cms (46.33%) with the mean height of the subjects being 166.08 ±8.48 cms. There was an increase in DML as the height increased. This rise was significant (p<0.05) in the ulnar nerve. CMAP amplitude showed a non-significant decrease as the heights increased whereas SNAP amplitude depicted a significant decrease in the median and ulnar nerves with an increase in height. Motor CV showed a significant (p<0.05) decrease as the height advances in ulnar nerves whereas in other nerves this decrease is non-significant. Sensory CV showed no significant decrease with an increase in height in the median and ulnar. F-wave minimum latencies showed a significant positive association with height, i.e., as the height increased F-wave minimum latency also increased. Thus we observed a negative association of CV and amplitude and a positive association of DML and F-wave minimum latency with height as shown in Table 5. Our observations were in accordance with findings by Soudmand et al. [4], who found an inverse correlation of CV with height (r=-0.46 (p<0.01); r=-0.36 (p<0.05)); a positive correlation between height and median, peroneal F-wave minimum latency (r=0.74 and 0.69, respectively; p<0.001). However, they observed no relationship between height and median (motor and sensory) CV (r=-0.04 (p>0.25);r=-0.14(p>0.25)), which is in contrast to our observation.

In the present study, the majority of subjects were with normal BMI 18.5-24.9 kg/m2 (69.11%) with the mean BMI of the subjects being 20.76 ±2.92 Kg/m2. No observable fixed trend could be seen in regard to DML and F-wave minimum latencies varying BMI. (Table 6) Only the median nerve was found to be associated with a lower amplitude (sensory and motor). Table 6 shows a non-significant slowing of both motor and sensory CV with increasing BMI. Our findings are in line with those of Awang et al. [14], who found a decrease in a CV in the median nerve (motor and sensory) and the ulnar with increasing BMI (motor). Despite this, they found no discernible change in the ulnar nerve's sensory properties. In motor ulnar and peroneal nerves, Buschbacher et al. [15] found a longer latency association with lower BMI. They also reported a significant association between latency and BMI in sensory radial and ulnar nerves. In all other sensory nerves and in the median nerves, no statistically significant difference was observed in latency, amplitude, and CV with varying BMI. In all sensory nerves, the amplitude was found to be varying with BMI (increased BMI association with lower amplitude).

## Conclusions

The present study concludes that age and height negatively influence amplitude and CV, whereas they positively influence DML and F-min latency. Significantly longer DML was observed as the age advances, except for ulnar nerve DML. There was an apparent decline in CMAP and SNAP amplitude. The reduction is significant in the motor median nerve, and slowing of motor and sensory CV was observed in the older age group that is significant in sensory ulnar and sensory median nerves. This rise was significant in the ulnar nerve. SNAP amplitude depicted a substantial decrease in the median and ulnar nerves with an increase in height. Motor CV showed a significant reduction as the height advances in the ulnar nerves. We observed a negative association between CV and amplitude and a positive association between DML and F-wave minimum latency with height. Higher BMI has a negative influence on amplitude and CV.
